# Setting up a three‐stage pre‐endoscopy triage during the coronavirus disease 2019 pandemic: A multicenter observational study

**DOI:** 10.1002/deo2.159

**Published:** 2022-08-08

**Authors:** Tao‐Chieh Liu, Chen‐Ling Peng, Fang‐Yu Hsu, Li‐Chun Chang, Hsiu‐Po Wang, Wei‐Kuo Chang

**Affiliations:** ^1^ Department of Internal Medicine Division of Gastroenterology Tri‐Service General Hospital, National Defense Medical Center Taipei Taiwan; ^2^ Department of Integrated Diagnostics and Therapeutics National Taiwan University Hospital, National Taiwan University College of Medicine Taipei Taiwan; ^3^ Department of Hepatology and Gastroenterology Chang Gung Memorial Hospital, Linkou Medical Center Taipei Taiwan; ^4^ Department of Internal Medicine Division of Gastroenterology and Hepatology National Taiwan University Hospital, National Taiwan University College of Medicine Taipei Taiwan

**Keywords:** COVID‐19, endoscopy, personal protective equipment, polymerase chain reaction, triage

## Abstract

**Objectives:**

Between May and July 2021, the coronavirus disease 2019 (COVID‐19) pandemic led to a sharp surge in community transmission in Taiwan. We present a three‐stage restructuring process of pre‐endoscopy triage at the beginning of the pandemic, which can support urgent endoscopic procedures while protecting endoscopy staff.

**Methods:**

The pre‐endoscopy triage framework was set up with three checkpoints at the hospital entrance, outpatient department, and endoscopy unit, with a specific target patient population and screening methods. Relevant data included the number of endoscopic procedures performed, outpatient department visits, and performing screening methods such as temperature measurement, travel, occupation, contact, and clustering history checking, polymerase chain reaction assay, and rapid antigen test.

**Results:**

Forehead temperature measurement and verification of travel, occupation, contact, and clustering history provided rapid, easy, and early mass screening of symptomatic patients at the hospital entrance. During the pandemic, outpatient department visits and endoscopic procedures decreased by 37% and 64%, respectively. The pre‐endoscopy screening methods used displayed regional variations in COVID‐19 prevalence. Among 16 endoscopy units with a community prevalence of ≥ 31.04 cases per 100,000 residents, 12 (75%) used polymerase chain reaction assay and four (25%) used rapid antigen test to identify asymptomatic patients before endoscopy. Of 6540 pre‐endoscopy screening patients, 15 (0.23%) tested positive by laboratory testing. No endoscopy‐related nosocomial COVID‐19 infections were reported during the pandemic.

**Conclusions:**

We present a three‐stage pre‐endoscopy triage based on the local laboratory capacity, medical resources, and community prevalence. These measures could be useful during the COVID‐19 pandemic.

## INTRODUCTION

Since the outbreak of a novel severe acute respiratory syndrome coronavirus 2 (SARS‐CoV‐2) in Wuhan, China, the disease has spread rapidly.^1^ The World Health Organization declared coronavirus disease 2019 (COVID‐19) a worldwide pandemic on March 11, 2020.^2^ Digestive endoscopy procedures pose a risk of infection for healthcare workers due to the potential aerosol and fecal‐oral transmission of microbial agents from patients. Pombo et al. reported that two‐thirds of all nurses and half of all technicians were infected in the endoscopy unit during the COVID‐19 pandemic.^3^

**FIGURE 1:**
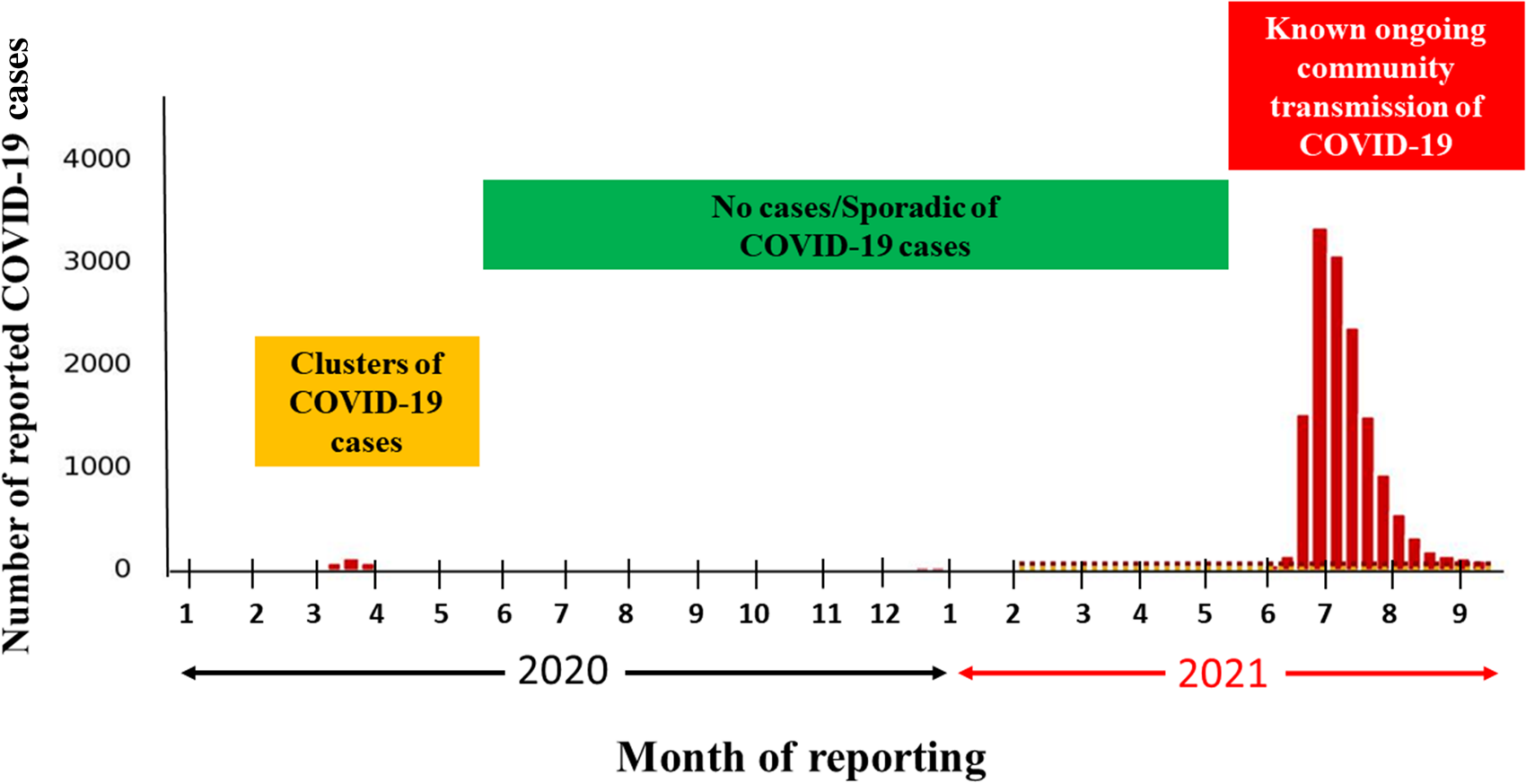
Number of COVID‐19 cases per month reported by Taiwan's Center for Disease Control. A small number of clusters of COVID‐19 cases were reported in early 2020, and there were no cases/sporadic cases of COVID‐19 reported in mid‐2020. A sharp surge in the community transmission was observed during May–July 2021. Abbreviations: COVID‐19, coronavirus disease 2019

The first confirmed case of COVID‐19 in Taiwan was announced on January 21, 2020.^4^ A small number of clusters of COVID‐19 cases were reported in early 2020 in Taiwan (Figure 1).^5^ In May 2021, a sharp surge began in the community transmission of the disease. Amid a sudden rise in the number of COVID‐19 cases, endoscopy units faced uncertainty about their functioning during the pandemic and about how the worst of the pandemic would end. Inefficient functioning of pre‐endoscopy triage can lead to overcrowding in the endoscopy unit and situations in which the rational use of personal protective equipment (PPE) is challenged, jeopardizing the morale of the endoscopy unit staff.^6^, ^7^, ^8^

Pre‐endoscopy triage screening is already in place in many medical centers. However, to the best of our knowledge, there are no published reports on the three steps of pre‐endoscopy triage screening in a real‐world situation. There is a paucity of information on the pre‐endoscopy triage established according to the local laboratory capacity, medical resources, and regional variations in the community prevalence of COVID‐19.^3^, ^6^, ^9^, ^10^, ^11^

If the triage and management system is not efficient, this can lead to overcrowding in the endoscopy units, resulting in medical staff exhaustion, missed endoscopic procedures for the patient, and situations in which the use of PPE is challenged.^6^, ^7^, ^8^ During the sharp rise in the number of COVID‐19 cases across Taiwan, endoscopy unit team leaders were required to take action at the beginning of the pandemic. In this response, a new, simple, and rapid method for pre‐endoscopy triage screening was developed and implemented, setting up a community‐based systematic pre‐endoscopy triage system, and considering the real‐world situations of laboratory testing capacity, medical resources, and community prevalence.

This study presents the pre‐endoscopy triage implemented in response to the COVID‐19 pandemic threat, which can support the need for urgent endoscopic procedures while protecting endoscopy staff.

## METHODS

### Study

This was a retrospective study of endoscopy units in referral medical centers that were members of the Digestive Endoscopy Society of Taiwan. We surveyed the restructuring of the pre‐endoscopy triage infection control process between May and July 2021, which represented the COVID‐19 pandemic period. The endoscopy units were surveyed between May and July 2020 corresponding to the period before the COVID‐19 pandemic. This study was approved by the Institutional Review Board of the Tri‐Service General Hospital, Taiwan.

### Three‐stage pre‐endoscopy triage

**FIGURE 2:**
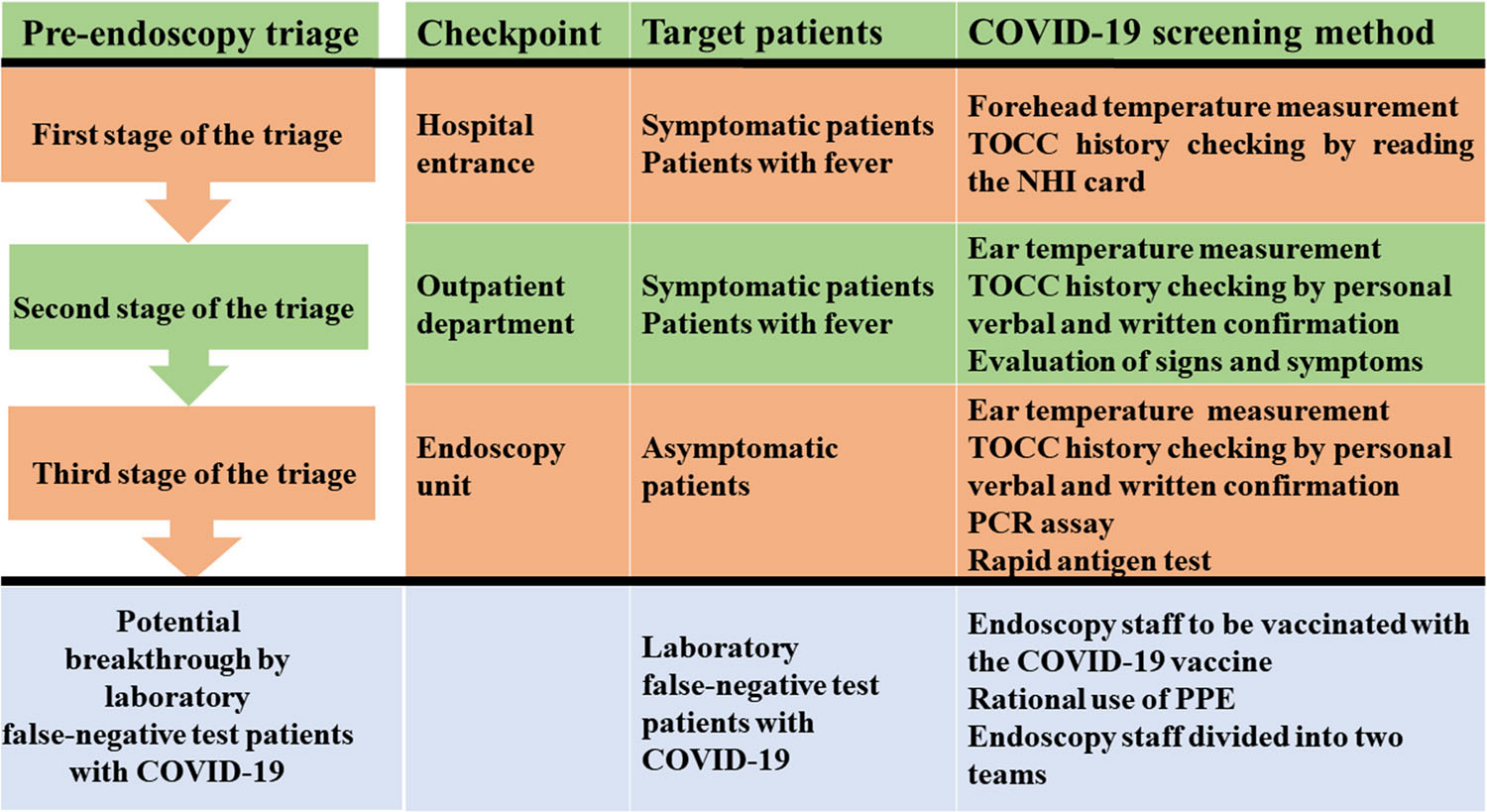
Framework of the three‐stage pre‐endoscopy triage which was set up using three checkpoints at the hospital entrance, outpatient department, and endoscopy unit. Abbreviations: COVID‐19, coronavirus disease 2019; PCR, polymerase chain reaction; TOCC, travel, occupation, contact, and cluster; PPE, personal protective equipment

The framework of the pre‐endoscopy triage (Figure 2) was set up with three checkpoints at the hospital entrance, outpatient department (OPD), and endoscopy unit, with a specific target patient population and screening methods.^6^, ^7^, ^8^, ^12^ Patients potentially infected with COVID‐19 were identified and redirected to a separate area by trained medical staff.

### Rationale of the pre‐endoscopy triage

#### First stage of the triage at the hospital entrance

In a report by the US Centers for Disease Control and Prevention, 370,000 confirmed COVID‐19 cases exhibited known symptoms such as fever (43%), cough (50%), and headache (34%).^13^ A study by Guan et al. reported that 1099 laboratory‐confirmed COVID‐19 cases presented common manifestations of COVID‐19 such as fever (88.7%), cough (67.8%), and fatigue (38.1%).^14^

**FIGURE 3:**
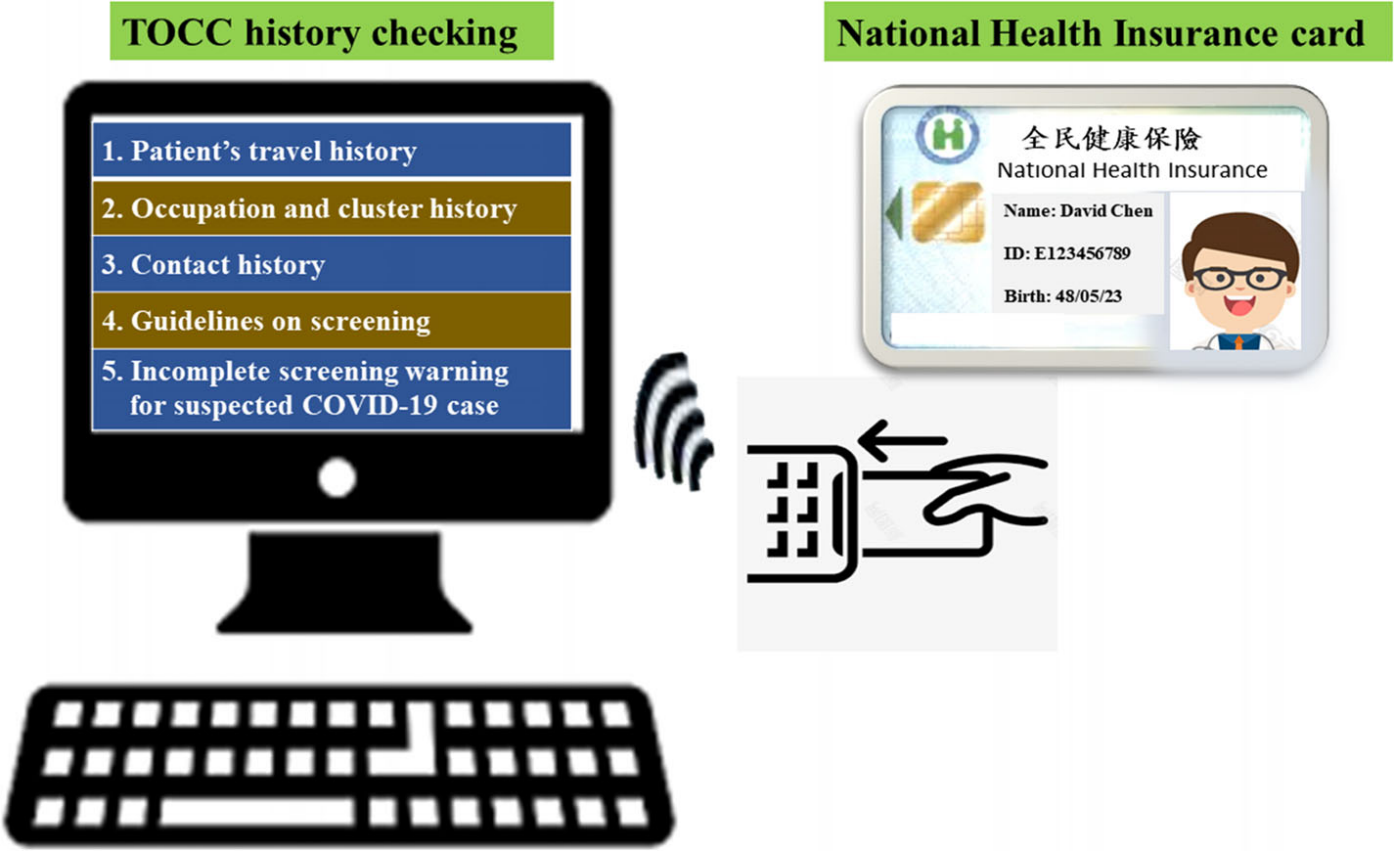
The National Health Insurance card can be used to confirm the TOCC history of the patients. The hospital staff can quickly obtain the patient's recent medical information, travel history such as travel to COVID‐19‐infected countries or regions, and can screen suspected warm patients during the COVID‐19 pandemic. Abbreviations: COVID‐19, coronavirus disease 2019; TOCC, travel, occupation, contact, and clustering

The objective of the first stage of the triage at the hospital entrance was to conduct rapid, easy, and early mass screening of people, with the aim of targeting the estimated 43%–88.7% of patients infected with COVID‐19 with fever and/or relevant travel, occupation, contact, and clustering (TOCC) history. Forehead temperature measurement and verification of TOCC history using the National Health Insurance (NHI) card (Figure 3) were conducted at the hospital entrance (Table 1). These efforts were undertaken to ensure rapid evaluation, prevent long waiting lines in front of the hospital, and prevent further nosocomial infections.

**TABLE 1:** Non‐laboratory‐based coronavirus disease 2019 (COVID‐19) screening methods

Screening method	Advantages	Disadvantages and limitations
**Temperature measurement**		
Forehead temperature	Quick measurement Easy for mass screening of patients Use of a non‐contact infrared thermometer	Variation in temperature during exercise, perspiration, wearing a head wrap, presence of sweat or dirt on the forehead
Ear temperature	Accurate measurement Core temperature can be measured	Disposable probe cover required Excess earwax or outer ear infection can affect the measurement Blood or drainage in the ear
**TOCC history checking**		
NHI card	Can quickly obtain patients’ medical information Visit COVID‐19‐infected country or region can be tracked	Does not comply with COVID‐19 preventive measures Does not report the traveling fingerprint
Survey questionnaire	Provides more accurate information	Time‐consuming and labor‐intensive
**Evaluation of patients**	Patient's signs and symptoms	Direct doctor‐patient contact

Abbreviations: COVID‐19, coronavirus disease 2019; NHI, National Health Insurance; TOCC, travel, occupation, contact, and cluster.

#### Second stage of the triage at the OPD

Forehead temperature measurement and verification of TOCC history based on the NHI card at the hospital entrance may not be sufficiently reliable (Table 1). The second stage of the triage at the OPD targeted symptomatic cases of COVID‐19 using ear thermometers to measure the temperature again, verifying TOCC history by seeking verbal and written confirmation from the patient, and assessing patients for clinical signs and symptoms. These efforts were undertaken to minimize the exposure of hospital staff and patients to COVID‐19, and to identify patients requiring laboratory‐based or non‐laboratory‐based screening for COVID‐19 before endoscopy.

#### Third stage of the triage at the endoscopy unit

Patients infected with COVID‐19 may be infectious for 1–3 days before symptom onset. Approximately 40%–50% of COVID‐19 cases are caused by transmission from asymptomatic or pre‐symptomatic patients.^15^, ^16^ The third stage of the triage at the endoscopy unit aimed to identify 40%–50% of asymptomatic patients with COVID‐19 using laboratory‐based screening methods such as the polymerase chain reaction (PCR) assay and rapid antigen test. The aim was to decrease the risk of nosocomial transmission of COVID‐19 and prevent systemic endoscopy unit shutdown.

#### Pre‐endoscopy triage screening methods for COVID‐19

The pre‐endoscopy triage screening methods for COVID‐19 included non‐laboratory‐based and laboratory‐based methods. The non‐laboratory‐based methods included (1) temperature measurement; (2) TOCC history checking; and (3) clinical evaluation of COVID‐19 at the OPD by clinical physicians (Table 1).^17^, ^18^, ^19^, ^20^ Laboratory‐based methods included the PCR assay and rapid antigen test.^6^, ^8^, ^21^, ^22^

#### Questionnaire survey

Two questionnaire surveys were administered to members of the Digestive Endoscopy Society of Taiwan. The first questionnaire survey of three major medical centers, located in a COVID‐19 hot spot area, included the Tri‐Service General Hospital, National Taiwan University Hospital, and Chang Gung Memorial Hospital with 1743, 2097, and 3406 hospital beds, respectively. The questionnaire survey included the following three categories: (1) number of digestive endoscopic procedures performed, including esophagogastroduodenoscopy, colonoscopy, endoscopic retrograde cholangiopancreatography, endoscopic ultrasonography, endoscopic mucosal resection, endoscopic submucosal dissection, and enteroscopy; (2) OPD visit volumes; and (3) pre‐endoscopy COVID‐19 screening methods.

The second questionnaire survey was conducted during the COVID‐19 pandemic. The questionnaire survey was delivered to 33 nationwide endoscopy units via e‐mail and a smartphone messaging application. A total of 29 endoscopy units (response rate of 88%) completed the survey. The questionnaire evaluating pre‐endoscopy screening methods included PCR assay, rapid antigen test, and verification of TOCC history.

## RESULTS

### Regional variations in the community prevalence of COVID‐19

**FIGURE 4:**
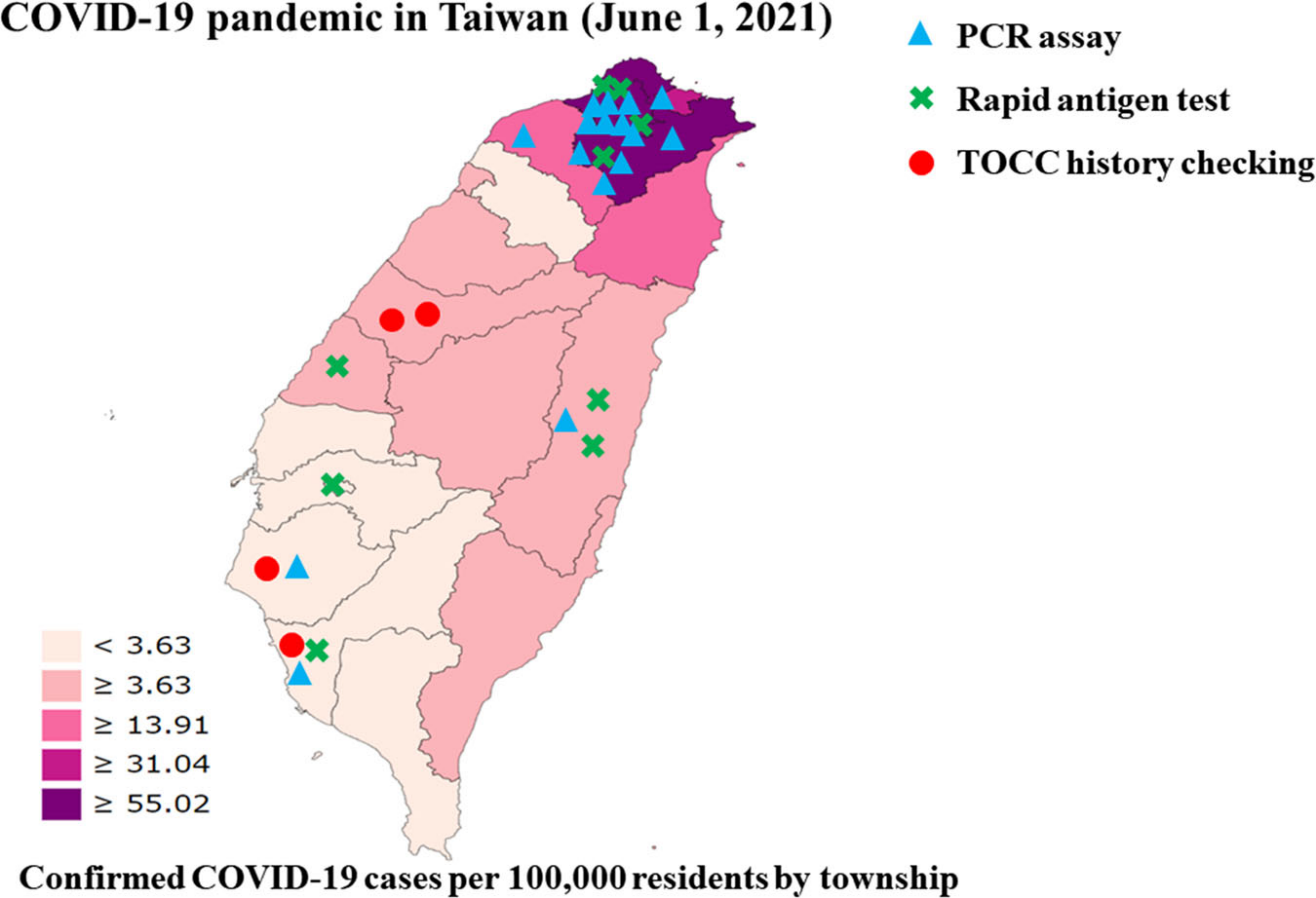
Regional variations in the pre‐endoscopy screening methods for COVID‐19 used by endoscopy units in Taiwan during the peak phase of the COVID‐19 pandemic (June 1, 2021). Northern Taiwan was the most affected during the COVID‐19 pandemic outbreak. Middle and Southern Taiwan had a low level of COVID‐19 community prevalence. The blue triangle (▲) denotes the PCR assay, the green cross (X) denotes the rapid antigen test and the red circle (●) denotes the TOCC history checking. Abbreviations: COVID‐19, coronavirus disease 19; PCR, polymerase chain reaction; TOCC, travel, occupation, contact, and clustering

In Taiwan, the number of confirmed COVID‐19 cases per 100,000 residents in the township was reported by the Taiwan Centers for Disease Control during the peak phase of the pandemic (June 1, 2021; Figure 4).^23^ During this period, regional variations in the community prevalence of COVID‐19 were observed. Northern Taiwan was the most affected part of the country during the outbreak, with a high community prevalence of ≥31.04 cases per 100,000 residents. Middle and southern Taiwan had a community prevalence of <31.04 confirmed cases per 100,000 residents.

### First stage of the triage at the hospital entrance

Information technology can improve the efficiency of triage screening. NHI enrolment is mandatory for all citizens and legal residents of Taiwan.^24^, ^25^ Patients visiting hospitals have to display their NHI card (Figure 3).^17^ Frontline hospital staff confirmed the medical information and TOCC history of patients by checking the NHI card and screened suspected febrile patients by conducting temperature measurements (Figure 2). Patients with fever or symptoms of COVID‐19 were taken care of in a separate area. No patient or visitor was allowed to enter the hospital building without wearing a surgical mask.

### Second stage of the triage at the OPD

**FIGURE 5:**
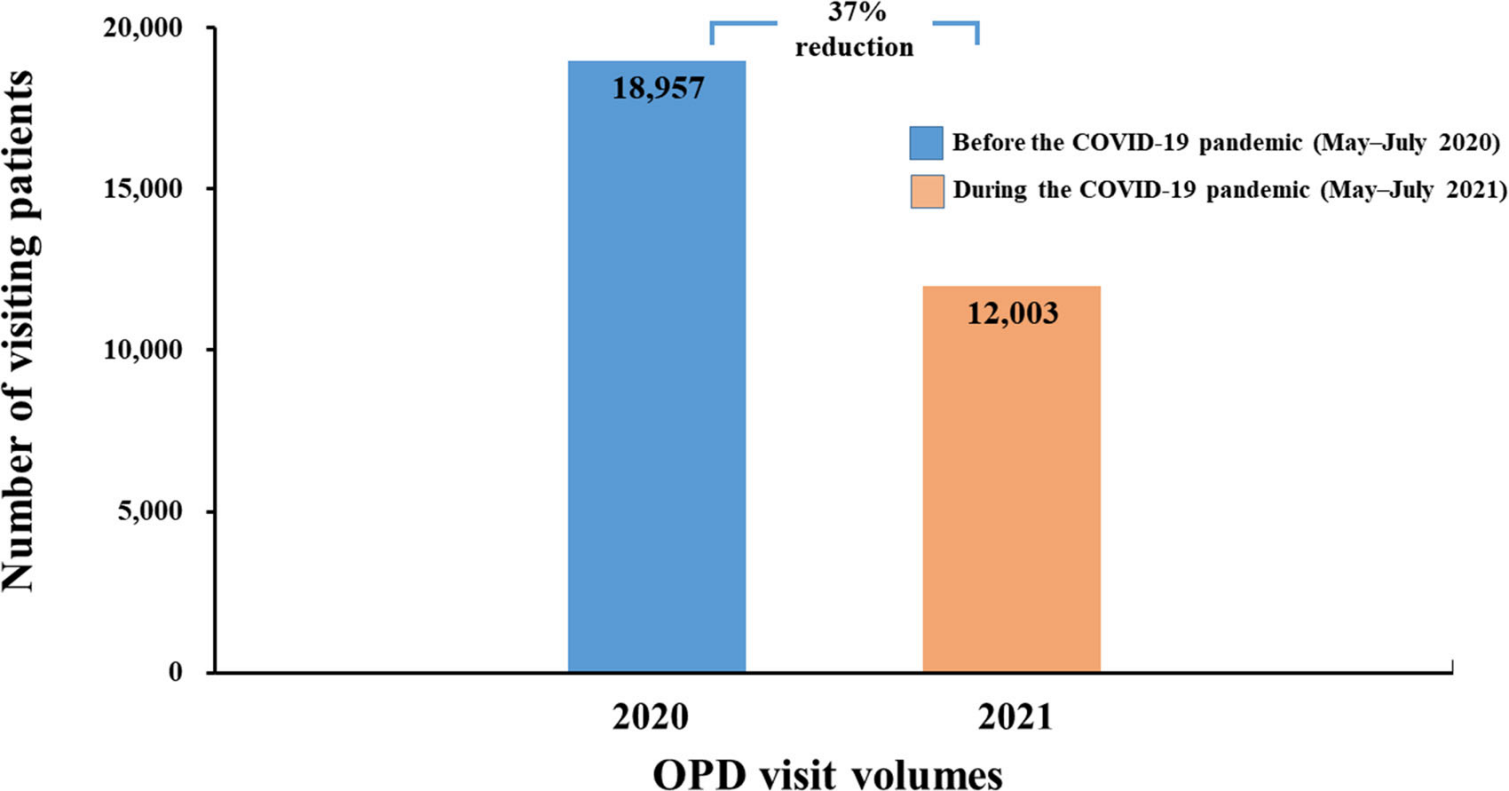
Comparison of the OPD visit volumes at the Tri‐Service General Hospital, Taiwan between two time periods: before the COVID‐19 pandemic (May–July 2020) versus during the COVID‐19 pandemic (May–July 2021). A 37% reduction in the OPD visit volumes was observed during May–July 2021. Abbreviations: COVID‐19, coronavirus disease; OPD, outpatient department

Gastrointestinal symptoms are common in patients with COVID‐19, and diarrhea, vomiting, abdominal discomfort, and loss of taste or smell occur in 26% of patients.^26^, ^27^, ^28^, ^29^ Patients who exhibited symptoms suggestive of COVID‐19 were redirected to a separate area. The following were conducted in all patients: repeat temperature measurement by measuring ear temperature, verification of TOCC history by seeking verbal and written confirmation from the patient, and examination of clinical signs and symptoms by physicians at the OPD. Compared to the period between May and July 2020 (Figure 5), there was a 37% reduction (*n* = 18,957 vs. *n* = 12,003) in the OPD visit volumes during the COVID‐19 pandemic period between May and July 2021.

### Third stage of the triage at the endoscopy unit

**FIGURE 6:**
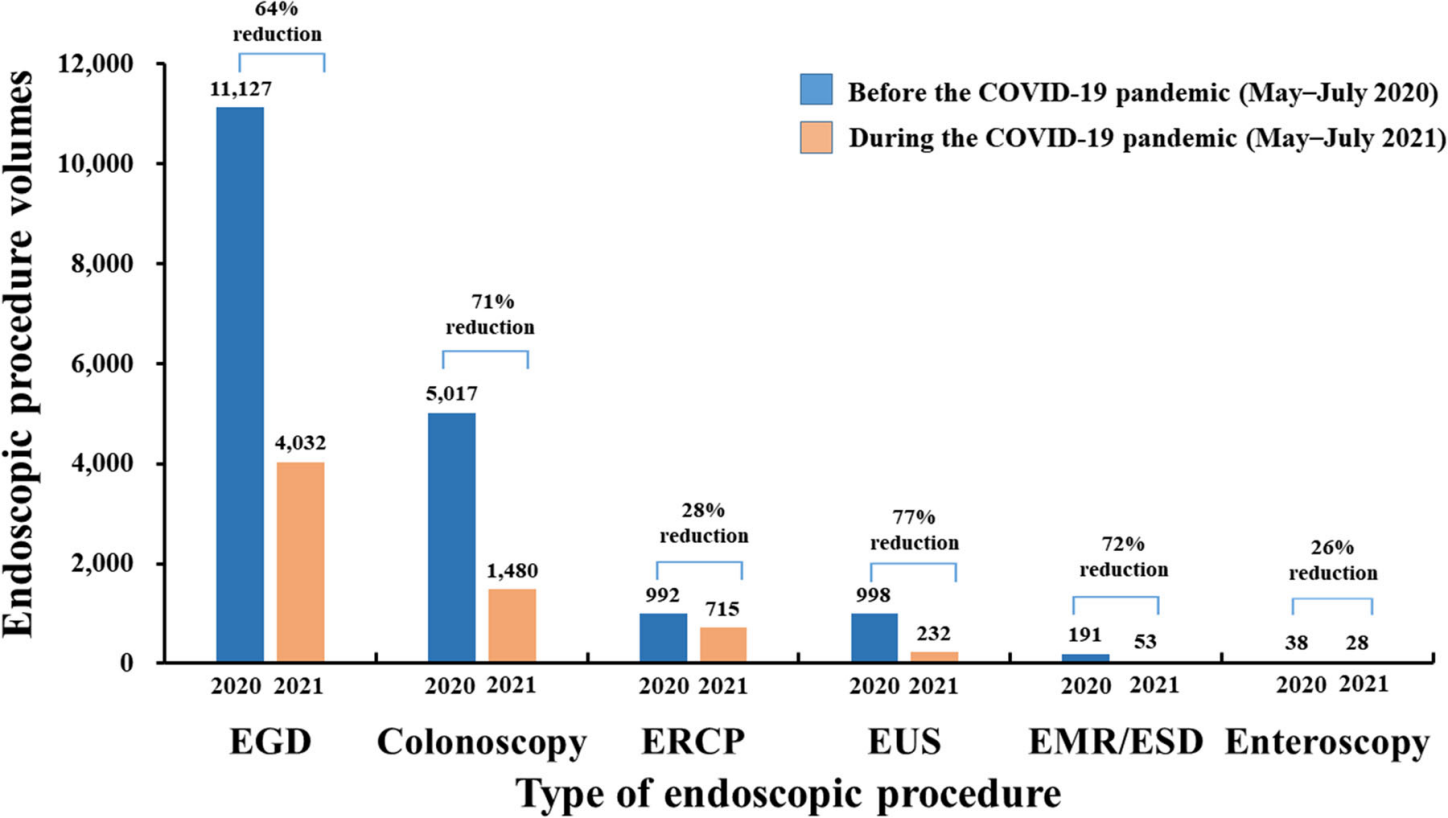
Comparison of the endoscopic procedure volumes between two periods: before the COVID‐19 pandemic (May–July 2020) versus during the COVID‐19 pandemic (May–July 2021). Data were obtained from the Tri‐Service General Hospital, National Taiwan University Hospital, and Chang Gung Memorial Hospital. Abbreviations: COVID‐19, coronavirus disease 2019; EGD, esophagogastroduodenoscopy; ERCP, endoscopic retrograde cholangiopancreatography; EUS, endoscopic ultrasonography; EMR, endoscopic mucosal resection; ESD, endoscopic submucosal dissection

A sharp rise in the number of COVID‐19 cases caused a temporary postponement of elective endoscopic procedures and non‐essential gastrointestinal office activities. During the COVID‐19 pandemic period (May–July 2021), there was a significant 64% reduction (*n* = 18,363 vs. *n* = 6540) in the total endoscopic procedure volume compared to the same period in 2020. The following reductions in procedure volumes were observed: 64% (*n* = 11,127 vs. *n* = 4032) for esophagogastroduodenoscopy, 71% (*n* = 5017 vs. *n* = 1480) for colonoscopy, 28% for endoscopic retrograde cholangiopancreatography (*n* = 992 vs. *n* = 715), 77% for endoscopic ultrasonography (*n* = 998 vs. *n* = 232), 72% for endoscopic mucosal resection/endoscopic submucosal dissection (*n* = 191 vs. *n* = 53), and 26% for enteroscopy (*n* = 38 vs. *n* = 28; Figure 6).

### Pre‐endoscopy screening methods for COVID‐19

A nationwide survey of endoscopy units was conducted during the COVID‐19 pandemic. We found that the PCR assay was costly (USD 32–67), time‐consuming (report time 6–12 h), and required a specialized laboratory setting. In contrast, rapid antigen tests present the following benefits to the healthcare system: decreased costs (USD 12–30) and rapid result turnaround time (2–4 h).

**FIGURE 7:**
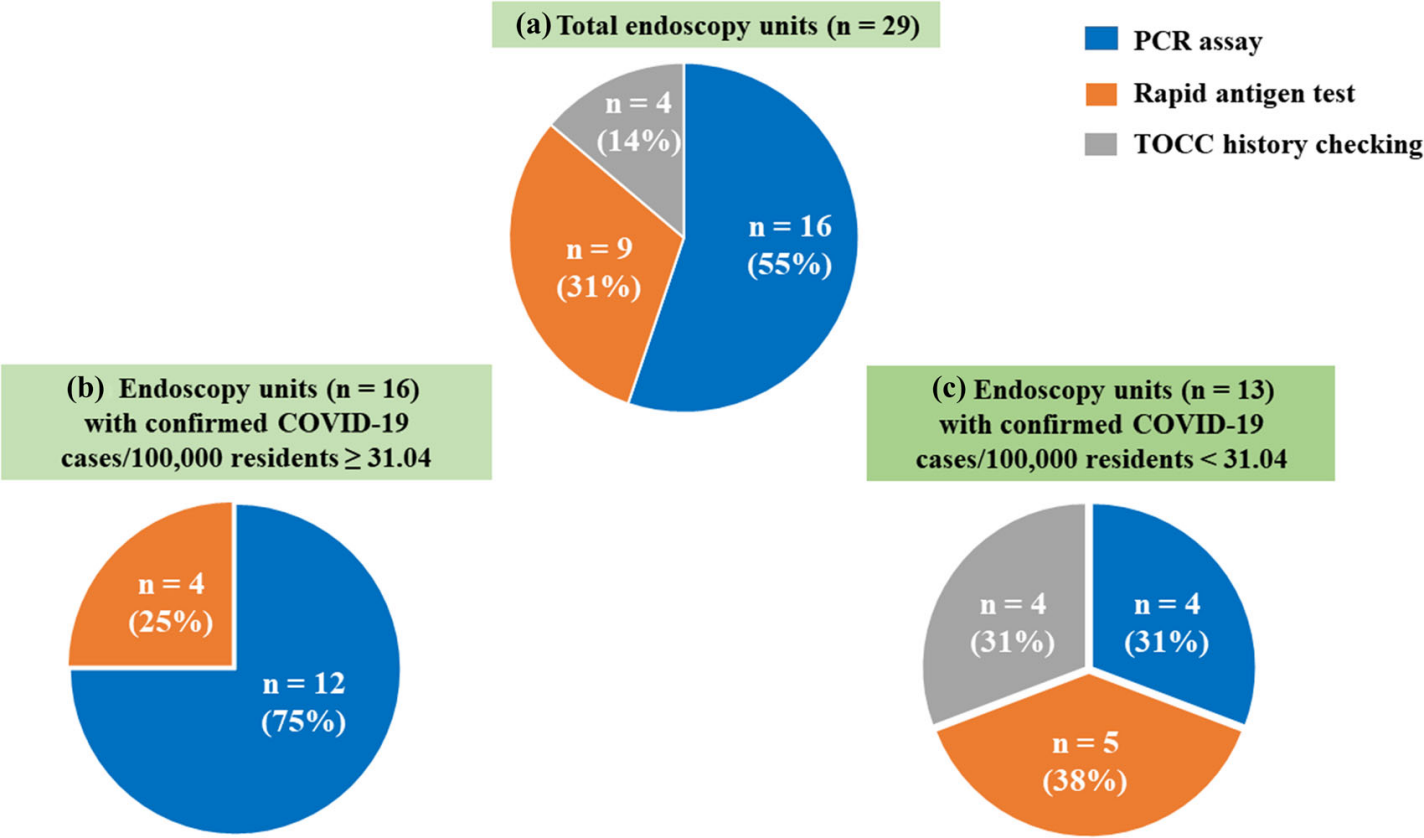
Pre‐endoscopy screening methods for COVID‐19 used at the endoscopy unit during the COVID‐19 pandemic. A total of 29 endoscopy units were surveyed (a), of which 16 had a COVID‐19 community prevalence ≥31.04 COVID‐19 cases per 100,000 residents (b), and 13 had a COVID‐19 community prevalence <31.04 COVID‐19 cases per 100,000 residents (c). Abbreviations: COVID‐19, coronavirus disease 2019; PCR, polymerase chain reaction; TOCC, travel, occupation, contact, and clustering

A total of 29 endoscopy units reported the use of pre‐endoscopy screening methods for COVID‐19, of which 16 (55%) used the PCR assay, 9 (31%) used the rapid antigen test, and 4 (14%) used the TOCC history checking (Figure 7a). During the COVID‐19 pandemic period, the COVID‐19 screening methods used displayed regional variations. The third stage of the triage using PCR and rapid antigen tests should be performed for all outpatients, particularly for the endoscopy units located in COVID‐19 hotspot areas (Figure 7b). Among 16 endoscopy units with a community prevalence ≥31.04 confirmed COVID‐19 cases per 100,000 residents (Figure 7b), 12 (75%) used the PCR assay and 4 (25%) used the rapid antigen test. Among 13 endoscopy units with a low‐level community prevalence of <31.04 confirmed COVID‐19 cases per 100,000 residents (Figure 7c), 5 (38%) used the rapid antigen test, 4 (31%) used the PCR assay, and 4 (31%) used the TOCC history checking.

### Diagnostic performance of laboratory‐based screening methods for COVID‐19

Of 6540 pre‐endoscopy screening patients, 15 (0.23%) tested positive for COVID‐19 based on laboratory‐based screening methods at the three participating hospitals. The PCR assay was positive in nine of 4819 patients (0.19%), and the rapid antigen test was positive in six of 1721 patients (0.35%) before endoscopy. No endoscopy‐related nosocomial COVID‐19 patient infections were reported during the pandemic.

### Potential breakthrough by laboratory false‐negative test patients with COVID‐19

In case of potential breakthrough infection by laboratory false‐negative test patients with COVID‐19, the endoscopy unit staff followed standardized precautions with the use of PPE for optimal COVID‐19 infection control. Furthermore, all staff were vaccinated with the first dose of the COVID‐19 vaccine and underwent PCR testing before the end of May 2021.

The staff of all endoscopy units (physicians, fellows, nurses, technicians, and staff) was divided into two teams. This ensures continued operation of an endoscopy unit even if one working group becomes incapacitated, preventing the systemic shutdown of the endoscopy unit.^30^ There were no endoscopy‐related COVID‐19 nosocomial infections reported during the COVID‐19 pandemic period in Taiwan.

## DISCUSSION

During the sharp rise in the number of COVID‐19 cases across Taiwan, endoscopy unit team leaders were required to optimize the protection, safety, and morale of the endoscopy unit staff, while responding to urgent endoscopic procedures.

### Forehead temperature measurement

Early identification of febrile patients can control the rapid spread of COVID‐19.^31^ Forehead temperature measurement was conducted at the hospital entrance (Table 1 and Figure 2). Infrared thermometers provide an easy, quick, non‐invasive, and non‐contact method of mass screening body temperature.^32^, ^33^

### Repeat temperature measurements using ear temperature

A minority of febrile patients may not be identified by the forehead thermometer during screening at the hospital entrance.^19^ Forehead skin temperature can vary by several degrees depending on exercise, perspiration, wearing a head wrap, presence of sweat or dirt on the forehead, or direct heat and air conditioning (Table 1).^34^ Ear thermometers can provide an accurate reflection of the patient's core temperature at the OPD and endoscopy unit.^35^

### TOCC history based on the NHI card

The NHI administration has contracted with 100% of hospitals, 92% of primary clinics, and 80% of pharmacies. Checking the NHI card at hospital arrival assists in obtaining patient medical information and travel fingerprints to COVID‐19‐infected countries or regions, and screening suspected febrile COVID‐19 patients (Table 1 and Figure 2).^20^, ^36^

### TOCC double‐checking by personal verbal and written confirmation

Patients may not comply with public health preventive measures and may not report their travel history. Verification of the TOCC history by verbal and written confirmation from the patient is warranted (Figure 2). TOCC screening forms were integrated into the electronic health record for TOCC standardized screening. This study demonstrated a 37% reduction in OPD visits (Figure 5) and a 64% reduction in endoscopic procedures during the COVID‐19 pandemic period (May–July 2021; Figure 6).

### Laboratory‐based screening methods for COVID‐19

Non‐laboratory‐based screening methods will fail to detect at least 50% of asymptomatic patients with COVID‐19. With the development of laboratory technology, pooling samples for the PCR assay is a cost‐ and time‐saving approach.^37^ Many endoscopy units could increase their laboratory capacity, improve the feasibility of testing, decrease patient burden, and avoid delays in time‐sensitive procedures.^37^

Rapid antigen tests can contribute to the overall COVID‐19 testing capacity, offering advantages in terms of shorter turnaround times and reduced costs, particularly in situations in which PCR testing capacity is limited.^21^, ^22^ The rapid antigen test is easily usable as a screening strategy prior to endoscopic procedures for the prevention of COVID‐19 transmission.^11^ The rapid antigen test may suffice because PCR testing for all patients involves a considerable cost burden and may delay endoscopy if PCR test results are pending.

### Endoscopy staff vaccination and PPE use

Approximately 0.1%–0.3% of laboratory false‐negative test asymptomatic patients with COVID‐19 may enter the endoscopy unit, resulting in potential nosocomial infection of COVID‐19 during the COVID‐19 pandemic.^8^ Endoscopy practice is conducted and supported by multiple endoscopy staff including physicians, nurses, technicians, assistants, secretaries, receptionists, janitors, and cleaners. Endoscopy staff were prioritized for COVID‐19 vaccine access and underwent PCR testing before the end of May 2021. It is compulsory for all staff members to undergo a body temperature measurement at the hospital entrance before work and to be monitored for COVID‐19‐associated symptoms every day. The endoscopy unit staff members wore a disposable waterproof surgical gown, gloves, shoe cover, face shield, and an N95 mask during endoscopic procedures.^38^, ^39^ High‐level PPE was preserved and used if the patient was COVID‐19 positive.^39^

### Limitations

This study has several limitations. First, a low number of patients tested positive (*n* = 15) for COVID‐19 by laboratory‐based screening methods. Second, our data could not determine the relative contribution of each component of the pre‐endoscopy triage in reducing the risk of endoscopy‐related transmission of infection. The combined effect of all these measures was beneficial and should be continued throughout the COVID‐19 pandemic. Third, the survey was conducted among local endoscopy units, and could not be distributed to all endoscopy units across the country. Hence, this study's findings may not be generalizable to all endoscopy units. The number of hospital beds at the Tri‐Service General Hospital, National Taiwan University Hospital, and Chang Gung Memorial Hospital was 1743, 2097, and 3406, respectively. Figures 5 and 6 show endoscopic practice‐derived high‐volume institutions located in a COVID‐19 hotspot area. This study did not evaluate the endoscopic practice obtained from the low‐volume institutions.

## CONCLUSIONS

This study was conducted among local endoscopy units and could not be distributed to all endoscopy units across the country. Modification and implementation of the pre‐endoscopy triage should be based on local laboratory infrastructure, test feasibility, medical resources, perceived burden on patients, and community prevalence of COVID‐19. These measures can be useful in preparing for future pandemics, as well as for a possible second wave of COVID‐19.

## CONFLICT OF INTEREST

The authors declare no conflict of interest.

## FUNDING INFORMATION

We are grateful for the financial support provided by the Ministry of National Defense Medical Affairs Bureau, Tri‐Service General Hospital, Taiwan (TSGH‐D‐111080 and TSGH‐D‐111081) for this study.
